# Serum vitamin D status inversely associates with a prevalence of severe sarcopenia among female patients with rheumatoid arthritis

**DOI:** 10.1038/s41598-021-99894-6

**Published:** 2021-10-14

**Authors:** Hiroto Minamino, Masao Katsushima, Mie Torii, Wataru Yamamoto, Yoshihito Fujita, Kaori Ikeda, Emi Okamura, Kosaku Murakami, Ryu Watanabe, Koichi Murata, Hiromu Ito, Masao Tanaka, Hidenori Arai, Shuichi Matsuda, Akio Morinobu, Nobuya Inagaki, Motomu Hashimoto

**Affiliations:** 1 Department of Diabetes, Endocrinology, and Nutrition, Graduate School of Medicine, Kyoto University, Kyoto, Japan; 2 Japan Society for the Promotion of Science, Tokyo, Japan; 3 Department of Rheumatology and Clinical Immunology, Graduate School of Medicine, Kyoto University, Kyoto, Japan; 4 Department of Human Health Sciences, Graduate School of Medicine, Kyoto University, Kyoto, Japan; 5 Department of Health Information Management, Kurashiki Sweet Hospital, Okayama, Japan; 6 Department of Advanced Medicine for Rheumatic Diseases, Graduate School of Medicine, Kyoto University, Kyoto, Japan; 7 Department of Clinical Immunology, Graduate School of Medicine, Osaka City University, Osaka, Japan; 8 Department of Orthopaedic Surgery, Graduate School of Medicine, Kyoto University, Kyoto, Japan; 9 Department of Orthopaedic Surgery, Kurashiki Central Hospital, Okayama, Japan; 10 National Center for Geriatrics and Gerontology, Aichi, Japan

**Keywords:** Rheumatology, Risk factors

## Abstract

Sarcopenia is an age-related disease with an increased risk of mortality. It is emerging that low serum 25-hydroxyvitamin D [25(OH)D] affects the sarcopenic state in general, but in rheumatoid arthritis (RA), these associations are not understood although the prevalence of vitamin D insufficiency is high in RA. We conducted a cross-sectional study of older female outpatients from our cohort (KURAMA) database. We measured skeletal muscle mass, handgrip strength, and gait-speed to diagnose severe sarcopenia. The serum 25(OH)D concentration was measured using electrochemiluminescence immunoassay. A total of 156 female patients with RA (sarcopenia:44.9%, severe sarcopenia: 29.5%, and without sarcopenia: 25.6%) were enrolled. Classification of vitamin D status at a cutoff point of median 25(OH)D concentration revealed that low 25(OH)D status was associated with a high prevalence of severe sarcopenia and with low measured values of muscle mass, handgrip, and gait speed. Furthermore, multivariable logistic regression analysis identified that low 25(OH)D status was associated with a high prevalence of severe sarcopenia (OR 6.00; 95% CI 1.99–18.08).The same association was observed when the cut-off value was set at 20 ng/ml. In components of sarcopenia, both low physical performance and muscle mass were associated with low 25(OH)D status. In conclusion, vitamin D status was inversely associated with severe sarcopenia, low physical performance, and low skeletal muscle mass. Modification of vitamin D status including vitamin D supplementation should be investigated as a therapeutic strategy for sarcopenic patients with RA.

## Introduction

Sarcopenia is an age-related disease characterized by poor physical performance and reduced muscle mass and strength, and one of the most important and challenging problems in an aging society. Sarcopenia has multifactorial etiology including malnutrition, aging, infrequent exercise and diseases such as cancer, diabetes mellitus, COPD and autoimmune diseases^1^; it is closely related to increased risk of mortality^2,3^. Although some therapeutic interventions are established such as encouragement of protein intake and physical exercise in the general population^4,5^, there still remain unknown aspects of sarcopenia in patients with debilitating diseases.

RA is an inflammatory autoimmune disease characterized by increased morbidity due to joint destruction together with extra-articular manifestations and increased mortality. RA patients exhibit far more sarcopenia than the general population^6^ and loss of physical performance such as walking and grip strength is a well-known risk factor of mortality^7^. Severe sarcopenia, which requires more intensive interventions to achieve improvements in physical performance^8,9^, is highly prevalent in RA patients compared to the general populations^10,11^. There have been conflicting results as to whether Disease Modifying Anti-Rheumatic Drugs (DMARDs) and biological agents improve the sarcopenic state in RA patients^10,12^; there is also little evidence on co-adjuvant therapies such as nutritional interventions in these patients.

Recently, vitamin D supplementation is receiving much attention as a potential therapeutic intervention for preventing sarcopenia in the general population together with protein supplementation and exercise. Vitamin D may well ameliorate the sarcopenic state via its role in muscle cell regulation, anti-inflammatory pathways and/or immunomodulatory responses^13^, as vitamin D status is associated with muscle strength and physical performance^14,15^. Indeed, meta-analyses and RCTs have shown that vitamin D supplementation improves limb strength in the community-dwelling elderly^16,17^. Interestingly, over 70% of RA patients exhibit vitamin D insufficiency^18^; these findings suggest the importance of adequate vitamin D provision on sarcopenia in RA populations as in general populations. However, it remains unclear whether serum vitamin D concentration affects the sarcopenic state in RA patients.

To elucidate the clinical association between vitamin D deficiency and sarcopenia in elderly patients with RA, we performed a cross-sectional study using the cohort study database, KURAMA cohort, established in 2011 for the storage of clinical data and specimens obtained from RA patients.

## Methods

### Study design and participants

We conducted a cross-sectional study in female RA outpatients from the Kyoto University Rheumatoid Arthritis Management Alliance (KURAMA) cohort database, which has been described in detail elsewhere^19,20^. We recruited older RA participants who visited the Kyoto University Hospital from May 2014 to December 2014 and who were over 60 years of age. All participants fulfilled the diagnostic criteria of the ACR/EULAR RA classification^21^. Data for 156 out of 248 participants was subjected to the planned analyses. 10 were excluded because of incomplete data set (lack of preserved serum or parameters of sarcopenia-related factors) and 82 were excluded because of the use of vitamin D supplementation (calcitriol such as eldecalcitol and alfacalcidol).

### Ethics

The study protocol and procedures were approved by the Medical Ethics Committee of Kyoto University Graduate School and Faculty of Medicine (Approval number: R0357) and complied with the principles of the Declaration of Helsinki. We obtained written informed consent was obtained from all patients of this study, which included the use of human blood samples and data.

### Diagnosis of sarcopenia and estimation of the related parameters

We measured muscle mass, muscle strength and physical performance to assess sarcopenia status in the study population, as previously described^10,22^. In brief, skeletal muscle mass was measured by bioelectrical impedance analysis (BIA) (Inbody 720: Biospace Co., Ltd., Seoul, Korea). Skeletal muscle index (SMI) was computed from the limb skeletal muscle mass in kilograms divided by the square of height in meters (kg/m^2^). Handgrip strength was measured using JAMAR digital hand dynamometer (Patterson Medical, Bolingbrook, IL). Gait speed was also measured by the 6-m walking speed using a portable gait rhythmogram (MG-M1110: LSI Medience Co., Tokyo, Japan) Regarding muscle strength and gait speed, the mean value of duplicate measurements was used for analysis.

Diagnosis of sarcopenia and severe sarcopenia were based on the criteria of the Asian Working Group for Sarcopenia (AWGS) 2019^23^. Briefly, sarcopenia was defined as low muscle mass with low muscle strength or with low physical performance; severe sarcopenia was defined as low muscle mass with both low muscle strength and low physical performance. Cut-offs values for low muscle mass, low muscle strength, and low physical performance were SMI < 5.7 kg/m^2^, handgrip strength < 18 kg, and gait speed < 1.0 m/s, respectively.

### Evaluation of 25(OH)D concentration and the clinical parameters

Serum 25(OH)D, an established biomarker reflecting vitamin D status, was measured using electrochemiluminescence immunoassay (LSI Medience Co., Tokyo, Japan). This laboratory used an external quality control provided by DEQAS , and the intra-assay CV varied between 2.64 and 5.65% across the range of 25(OH) concentration, between 12.87 and 59.16 ng/ml. MNA-SF (Mini Nutritional Assessment Short-Form) was collected to assess nutritional state^24^. RA disease activity and physical dysfunction were assessed by a 28-Joint RA Disease Activity Score (DAS28-ESR), the doctor or patient Visual Analogue Scale (Dr. or PT-VAS), Steinbrocker’s stage and class and the health assessment questionnaire disability index (HAQ). We also reviewed the information regarding current RA therapeutics including methotrexate, prednisolone, and biological agents (TNF inhibitors: n = 33, IL-6 receptor inhibitor: n = 12, CTLA4-immunoglobulin: n = 10). Other epidemiologic and anthropometric variables including age, duration of RA disease and body mass index (BMI) were extracted from the KURAMA database. Information on falls and fractures in the last year was collected from all subjects by a self-reported questionnaire form. The use of osteoporosis medication was obtained from the electronic medical record.

### Statistical analysis

We present continuous variables as the mean (standard deviation (SD)) or as the median (interquartile range (IQR)) and categorial variables as numbers (%). To compare baseline characteristics according to 25(OH)D status, we divided participants into the following two groups by the median of serum 25(OH)D concentration: lower status group (25(OH)D: 5.9–16.0 ng/ml); higher status group (25(OH)D: 16.1–32.1 ng/ml). We then performed a Mann–Whitney’s U test or a Fisher’s exact test for continuous variables and categorical variables, respectively. To explore the relationship between 25(OH)D status and severe sarcopenia, univariate and multivariate logistic regression analyses were conducted. 25(OH)D status was used as either a binary variable as described above, a binary variable with a cutoff value of 20 ng/ml, which value is used for an indicator of bone health^25^, or a continuous variable in multivariate analysis. In multivariate analyses, we constructed the following multiple models by incorporating significant variables in the univariate analysis and clinically relevant variables including RA therapeutics^10,26^: model 1 was adjusted for 25(OH)D status, age, and body mass index; model 2 was adjusted for variables in model 1 plus nutrition status (MNA-SF) and RA-related factors (DAS28-ESR, Stage 3 and 4 vs. 1 and 2, HAQ, and therapeutics (use of prednisolone, biologics, and methotrexate)); model 3 was adjusted for variables in model 2 plus the prevalence of osteoporosis medication, as RANKL inhibitors can exert a positive effect on muscle strength^27^. We also adopted multivariate logistic regression analysis using the same models for each component of severe sarcopenia (low muscle mass, low muscle strength, and low physical performance). JMP 15.2.0 (SAS Institute Inc., Cary, NC, USA) was used for statistical analyses; a value of *P* < 0.05 was considered significant.

## Results

Participant characteristics of this study are provided in Table 1. Data for 156 female RA patients with a mean (SD) age of 69.7 (6.7) was subjected to the following analysis. The median (IQR) serum 25(OH)D concentration was 16.0 (12.8–19.2), which represents a lower concentration than that in the general population^28,29^ and accords with that in other RA studies^18^. As for RA-related factors, the average (SD) duration of RA was 16.1 years (12.7). Disease activity of RA (DAS28-ESR) was generally low under the following therapeutics: methotrexate in 67.3%, prednisolone in 27.6%, and biological agent in 35.3%, but about two-thirds of the patients had advanced joint deformity or destruction as assessed by a Steinbrocker’s stage of 3 (21.1%) or 4 (46.8%).

**Table 1:** Characteristics of this study population.

Characteristics	RA patients
	(*N* = 156)
Age, mean (SD), years	69.7 (6.7)
**Body composition and physical activity variables**	
Body mass index, mean (SD), kg/m^2^	22.0 (3.6)
Skeletal mass index, mean (SD), kg/m^2^	5.64 (0.83)
Handgrip strength-dominant, mean (SD), kg	14.5 (7.2)
Gait speed, mean (SD), m/s	0.95 (0.29)
Sarcopenia (+), *n* (%)	70 (44.9)
Severe sarcopenia (+), *n* (%)	46 (29.5)
Any fall in the previous year, *n* (%)	25 (16.2)
Any fracture in the previous year, *n* (%)	7 (4.6)
Osteoporosis medication, *n* (%)	45 (28.9)
MNA-SF, mean (SD)	12.0 (2.0)
**RA disease characteristics**	
Duration, mean (SD), years	16.1 (12.7)
DAS28-ESR, mean (SD)	2.96 (0.98)
HAQ score, mean (SD)	0.83 (0.74)
Stage*, mean (SD)	3.01 (1.10)
Stage 1, *n* (%)	21 (13.4)
Stage 2, *n* (%)	29 (18.6)
Stage 3, *n* (%)	33 (21.1)
Stage 4, *n* (%)	73 (46.8)
Class*, mean (SD)	1.82 (0.60)
**Current RA medications**	
Methotrexate use, *n* (%)	105 (67.3)
Prednisolone use, *n* (%)	43 (27.6)
Biological agent use, *n* (%)	55 (35.3)
**Laboratory data**	
Serum 25(OH)D, median (IQR), ng/ml	16.0 (12.8–19.2)
CRP, median (IQR), mg/dL	0.1 (0.075–0.30)

Data are presented as the mean (standard deviation (SD)) or as the median (interquartile range (IQR)) for continuous variables, and as numbers (%) for categorial variables.

*RA* rheumatoid arthritis, *MNA-SF* Mini Nutritional Assessment Short-Form, *DAS28* disease activity score using 28 joints, *VAS* visual analogue scale, *HAQ* health assessment questionnaire.

*Steinbrocker's classification.

Of these participants, on the basis of the diagnostic criteria of sarcopenia in AWGS 2019^23^, 46 (29.5%) were determined to have severe sarcopenia and 70 (44.9%) were determined to have sarcopenia. Regarding the components of sarcopenia, mean (SD) of SMI, handgrip strength and gait speed were 5.64 (0.83), 14.5 (7.2) and 0.95 (0.29), respectively. Under these conditions, 50.6%, 67.3%, and 50.6% of the participants fulfilled the criterion for low muscle mass, low muscle strength (handgrip strength), or poor physical performance (gait speed), respectively.

### Comparison of clinical characteristics according to 25(OH)D status

To determine whether serum 25(OH)D status was associated with RA-related factors and sarcopenia in the RA population, we first divided participants into two groups (lower/higher) at a cut-off point of the median serum 25(OH)D concentration and then compared their characteristics. As for the components of sarcopenia, skeletal muscle mass, handgrip strength and gait speed were significantly lower in the low 25(OH)D group (Table 2). The prevalence of severe sarcopenia was significantly higher in the low 25(OH)D group, which was also in the case in sarcopenia. Regarding RA-related factors, as in other reports^30,31^, DAS28-ESR, the degree of current disease activity, tended to be higher in the low 25(OH)D group. In the lower 25(OH)D group, the % of patients on prednisolone use was significantly higher (35.9% vs. 19.2%).

**Table 2:** Characteristics of participants by serum 25(OH)D status.

25(OH)D concentration (range, ng/ml)	Lower status (*n* = 78)	Higher status (*n* = 78)	*P* value
	5.9–16.0	16.1–32.1	
Age, mean (SD), year	70.4 (6.9)	69.1 (6.4)	0.21
Body mass index, mean (SD), kg/m^2^	21.7 (3.5)	22.2 (3.6)	0.29
**Factors associated with sarcopenia**			
Skeletal mass index, mean (SD), kg/m^2^	5.45 (0.90)	5.83 (0.69)	**0.0036**
Handgrip strength-dominant, mean (SD), kg	13.1 (7.6)	16.0 (6.5)	**0.0094**
Gait speed, mean (SD), m/s	0.88 (0.30)	1.02 (0.27)	**0.0025**
Sarcopenia (+), *n* (%)	44 (56.4)	26 (33.3)	**0.0036**
Severe sarcopenia (+), *n* (%)	34 (43.6)	12 (15.4)	** < 0.0001**
Osteoporosis medication, *n* (%)	41 (35.0)	36 (29.8)	1.00
MNA-SF, mean (SD)	11.9 (2.1)	12.0 (1.9)	0.69
**RA disease characteristics**			
Disease duration, mean (SD), year	16.6 (13.6)	15.6 (11.7)	0.63
DAS28-ESR, mean (SD)	3.11 (1.04)	2.81 (0.90)	0.055
CRP, median (IQR), mg/dL	0.1 (0–0.4)	0.1 (0.1–0.3)	0.44
HAQ, mean (SD)	1.00 (0.79)	0.67 (0.66)	**0.0096**
Stage, mean (SD)	2.99 (1.10)	3.04 (1.10)	0.77
Stage 4, *n* (%)	35 (44.9)	38 (48.7)	0.63
Stage 3 and 4, *n* (%)	53 (68.0)	53 (68.0)	1.00
Stage 2, 3 and 4, *n* (%)	67 (85.9)	68 (87.2)	0.81
Class, mean (SD)	1.91 (0.65)	1.73 (0.53)	0.060
**Current therapeutic agent**			
Methotrexate use, *n* (%)	49 (62.8)	56 (71.8)	0.23
Biological agent use, *n* (%)	29 (37.2)	26 (33.3)	0.61
Prednisolone use, *n* (%)	28 (35.9)	15 (19.2)	**0.019**

RA patients are divided into the following two groups by median of serum 25(OH)D: lower status group (25(OH)D: 5.9–16.0 ng/ml) and higher status group (25(OH)D: 16.1–32.1 ng/ml). Data are presented as the mean (± standard deviation) or as the median (interquartile range (IQR)) for continuous variables, and as numbers (%) for categorial variables.

*RA* rheumatoid arthritis, *MNA-SF* Mini Nutritional Assessment Short-Form, *DAS28* disease activity score using 28 joints, *VAS* visual analogue scale, *HAQ* health assessment questionnaire.

### Low 25(OH)D status is independently and positively associated with a high prevalence of severe sarcopenia

Although it is emerging that low 25(OH)D concentrations affect the sarcopenia state in the general population^14,15^, in a RA population these associations are not well understood. We therefore performed univariate and multivariate regression analyses with the presence of severe sarcopenia as the dependent variable. Univariate logistic regression analyses revealed that low 25(OH)D status was significantly associated with a high prevalence of severe sarcopenia (OR 4.25; 95% CI 1.99–9.09) (Table 3 left). Age, BMI, DAS28-ESR, Stage, HAQ score, the use of prednisolone and nutritional status (MNA-SF) were also associated with severe sarcopenia. We then conducted multivariate logistic regression analyses to clarify whether low 25(OH)D status independently contributes to severe sarcopenia cross-sectionally. Among the factors that were significant in the univariate analysis, we excluded the HAQ score from covariates in the multivariate analyses because this score might be a result, not a cause, of sarcopenia. We then determined that low 25(OH)D status was independently associated with high prevalence of severe sarcopenia in model 1 adjusted for age, BMI, 25(OH)D status (OR 4.42; 95% CI 1.80–10.8) (Table 3 middle). In other models adjusted for RA-related and nutritional factors (model 2) or adjusted for factors that may affect 25(OH) D including osteoporosis medication (model 3), the same significant relationships still remained. When 25(OH)D was modeled as a continuous variable, lower 25(OH)D concentrations were also significantly associated with severe sarcopenia in all models (OR 0.91; 95% CI 0.84–0.99: model 1) (Supplementary Table S1). Similarly, when 25(OH)D was used as a binary variable with a cutoff value of 20 ng/ml, lower 25(OH)D was also significantly associated with severe sarcopenia in all models (OR 3.87; 95% CI 1.14–13.1: model 1) (Supplementary Table S2).

**Table 3:** Logistic analysis for RA patients with severe sarcopenia.

Variables	Univariate		Multivariate					
			Model 1		Model 2		Model 3	
	OR (95% CI)	*P* value	OR (95% CI)	*P* value	OR (95% CI)	*P* value	OR (95% CI)	*P* value
Age (1 year)	1.15 (1.08–1.22)	< 0.0001	1.17 (1.09–1.25)	< 0.0001	1.21 (1.10–1.33)	< 0.0001	1.21 (1.10–1.33)	< 0.0001
Body mass index (1 kg/m^2^)	0.76 (0.67–0.86)	< 0.0001	0.72 (0.62–0.84)	< 0.0001	0.76 (0.60–0.95)	0.0085	0.76 (0.60–0.95)	0.010
Low 25(OH)D status (≦16.0 ng/ml)	4.25 (1.99–9.09)	< 0.0001	4.42 (1.80–10.8)	0.0007	5.92 (1.98–17.7)	0.0006	6.00 (1.99–18.08)	0.0006
DAS28-ESR	1.58 (1.09–2.31)	0.015			1.03 (0.62–1.74)	0.90	1.23 (0.86–1.76)	0.88
Stage (3, 4 vs. 1, 2)	4.44 (1.74–11.4)	0.0005			4.33 (1.33–14.08)	0.0010	4.40 (1.35–14.32)	0.0097
HAQ	4.03 (2.19–7.41)	< 0.0001						
Methotrexate use	0.58 (0.28–1.19)	0.14			2.18 (0.66–7.22)	0.19	2.18 (0.66–7.22)	0.19
Prednisolone use	2.91 (1.39–6.11)	0.0049			2.45 (0.74–8.06)	0.09	2.45 (0.74–8.06)	0.13
Biological agents use	0.47 (0.21–1.03)	0.058			0.70 (0.22–2.21)	0.52	0.70 (0.22–2.21)	0.54
MNA-SF	0.70 (0.59–0.85)	< 0.0001			0.91 (0.66–1.24)	0.54	0.90 (0.66–1.24)	0.53
Osteoporosis medication (+)	1.72 (0.82–3.59)	0.15					1.22 (0.40–3.72)	0.73

Results of univariate (left) and multivariate (right) logistic analyses for independent variables associated with severe sarcopenia. Model 1 was adjusted for vitamin D status, age, and body mass index. Model 2 was adjusted for variables in model 1 plus nutrition status (MNA-SF), and RA-related factors (DAS28-ESR, Stage, HAQ, and therapeutics (use of prednisolone, biologics, and methotrexate)). Model 3 was adjusted for variables in model 2 plus the prevalence of osteoporosis medication.

*RA* rheumatoid arthritis, *DAS28* disease activity score using 28 joints, *HAQ* health assessment questionnaire, *MNA-SF* Mini Nutritional Assessment Short-Form.

### 25(OH)D status associates both with physical performance and skeletal muscle mass

**Figure 1:**
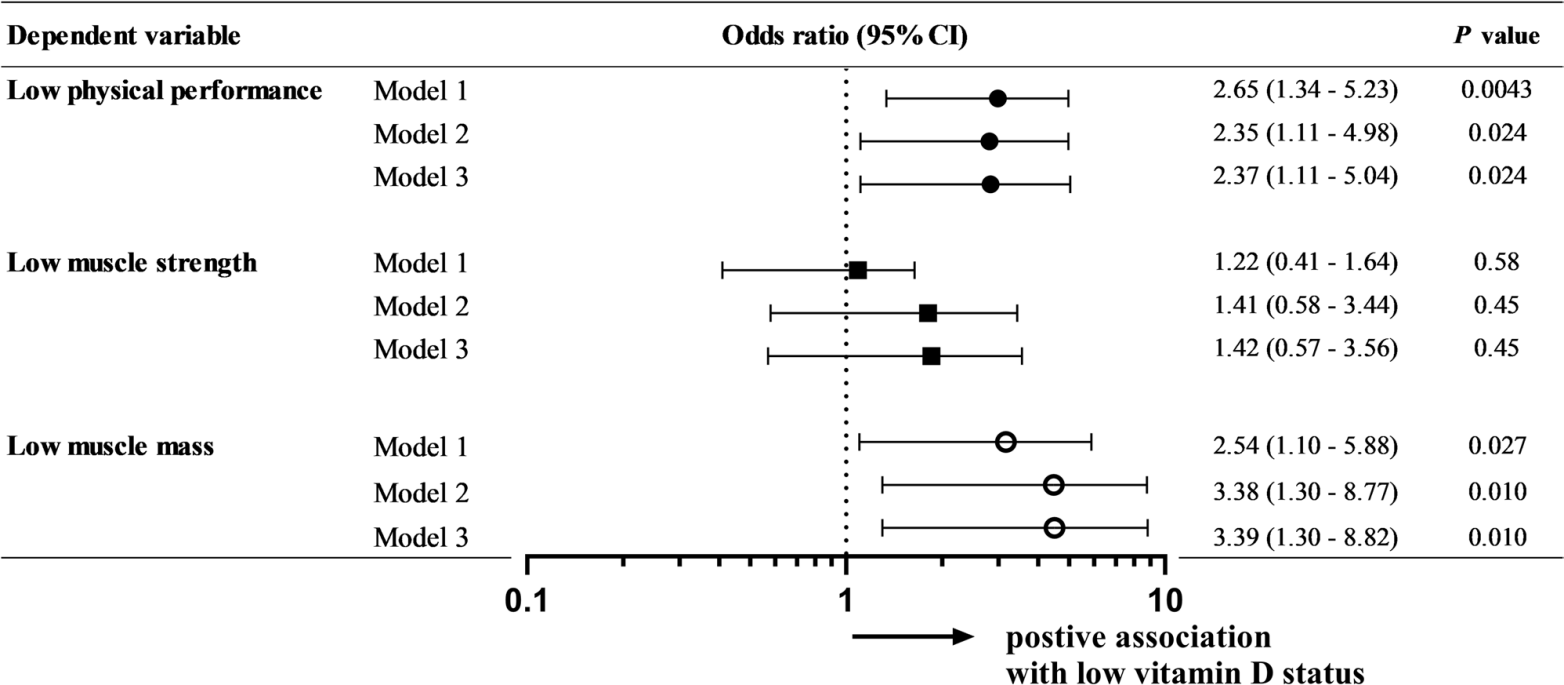
Associations between vitamin D status and components of sarcopenia. Results of multivariate logistic analyses for independent variables associated with components of sarcopenia. This forest plot represents the odds ratio and 95% confidence interval (CI) for each sarcopenia-related component in each adjusted model. Model 1 was adjusted for vitamin D status, age, and body mass index. Model 2 was adjusted for variables in model 1 plus nutrition status (MNA-SF) and RA-related factors (DAS28-ESR, Stage, HAQ, and therapeutics (use of prednisolone, biologics, and methotrexate)). Model 3 was adjusted for variables in model 2 plus the prevalence of osteoporosis medication.

We then conducted further multivariate analyses to clarify which components of severe sarcopenia were most strongly associated with low serum 25(OH)D status. Regarding the components of severe sarcopenia, both low physical performance and low skeletal muscle mass were significantly associated with low serum 25(OH)D status in model 1 (physical performance: OR 2.65; 95% CI 1.34–5.23, skeletal muscle mass: OR 2.54; 95% CI 1.10–5.88) (Fig. 1). The same associations were maintained in the other models (model 2 and 3). Other variables are described in supplementary Table S3. There was no significant association with muscle strength. Low serum 25(OH)D is therefore independently associated with a high prevalence of severe sarcopenia and with both low physical performance and low skeletal muscle index mass among the measures used to assess sarcopenia.

## Discussion

**Figure 2:**
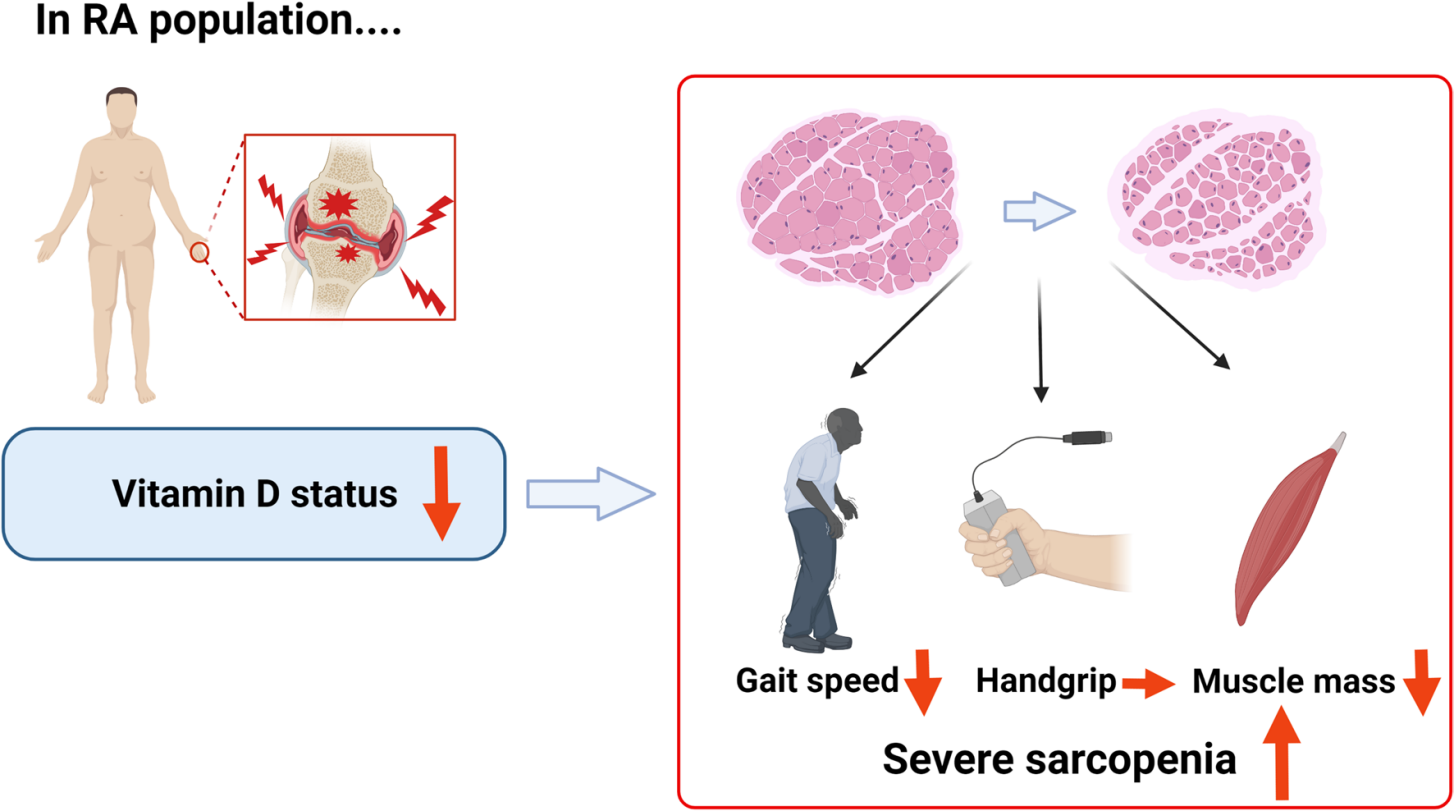
A proposed model of the relationship between vitamin D status, severe sarcopenia and its components in RA patients. In this study, vitamin D deficiency were strongly associated with increased prevalence of severe sarcopenia and impaired lower limb performance and skeletal muscle in RA patients. Modification strategy of vitamin D status including vitamin D supplementation may contribute to the improvement of sarcopenia in RA. This figure was created with BioRender.com (https://biorender.com/). *RA* rheumatoid arthritis.

The present study is the first to document a significant association between vitamin D deficiency and severe sarcopenia in a female RA population. Our participants had a high prevalence of severe sarcopenia (29.5%) and vitamin D deficiency (median 16.0 ng/ml); multivariate analyses showed low serum 25(OH)D to be an independent risk for severe sarcopenia (OR 4.42). In addition, among the three components of sarcopenia, decreased gait speed and muscle mass showed significant associations with low serum vitamin D. These findings accord with those in general population studies: low serum vitamin D has a stronger association with lower function on walking than on other physical performance or muscle strength^32,33^. Our results suggest that vitamin D deficiency may contribute to the development of severe sarcopenia and impaired lower limb performance and skeletal muscle in RA patients, as is the case in the general population (Fig. 2).

Recently, basic studies have provided insight into the mechanism of vitamin D action on skeletal muscle function, including modulation of muscle differentiation, oxidative stress, cellular metabolism and inflammatory condition^13,34^. The expression of vitamin D receptors in muscle nuclei decreases with aging, which affects muscle differentiation and is implicated in the development of sarcopenia^35^. Vitamin D deficiency also contributes to skeletal muscle atrophy via dysregulation of cellular metabolism such as oxidative stress and calcium homeostasis resulting in mitochondrial function^36,37^. Furthermore, vitamin D is well known for its anti-inflammatory and immunomodulatory properties^38^. It suppresses Th1, Th17 and macrophage cytokines (IL-2, IFN-γ, IL-17, IL-21, TNF-α, IL-6, IL1-β) as well as innate immune responses such as toll-like receptor signaling and antigen presentation^39,40^; it promotes the expression of Th2 cytokines (IL-4, IL-5, IL-10, IL-13) and the differentiation of regulatory T cells^41,42^. Given that Th1 and Th17 responses participate in the pathogenesis of RA^43^, vitamin D might well ameliorate sarcopenia in RA patients partly through attenuating chronic inflammation that results in muscle catabolism as well as through modulating muscle function via multiple physiological pathways^13,36,37^.

Similarly, epidemiological studies have shown clinical correlations between vitamin D deficiency and sarcopenia. In general populations, low serum vitamin D concentration significantly associates with increased prevalence of sarcopenia and loss of physical performance such as walking speed^14,15^. Several meta-analyses and RCTs have also shown the positive effects of vitamin D supplementation such as improvement in global muscle strength (especially lower limb muscles) and a decrease in the sit-to-stand time^16,17^. In RA populations, however, the relationship between sarcopenia and vitamin D status has not been clarified even though the prevalence of sarcopenia and vitamin D insufficiency is markedly higher in the RA population than it is in the general population^6,44^. The present study provides novel evidence that low serum 25(OH)D is a significant risk factor of severe sarcopenia in RA. Taking these findings together, vitamin D deficiency may well be a candidate co-adjuvant therapeutic target to prevent severe sarcopenia in RA patients; prospective studies of vitamin D supplementation to prevent sarcopenia in RA will be worthwhile.

There are several limitations in the present study. The present cross-sectional study does not imply causation and further prospective investigation is needed. There is also the possibility of selection bias because the study included only female patients older than 60 years, although these are the dominant populations with RA. We excluded vitamin D supplementation as a confounding factor in the present study, which might produce a biased group of subjects: our findings are applicable only to RA patients without supplementation therapy. In addition, there might remain unadjusted confounding variables related to vitamin D status and sarcopenia including dietary profile, seasonal variation and socioeconomic status, and information on falls and fractures was obtained by personal recall.

In conclusion, female RA patients with low serum 25(OH)D concentration has a significant risk for a higher prevalence of severe sarcopenia, low physical performance, and low skeletal muscle mass. The interventions to improve serum 25(OH)D concentration including vitamin D supplementation could be beneficial for RA patients with sarcopenia.

## Supplementary Information

Supplementary Information.
