# Supplementary material for: Serum vitamin D status inversely associates with a prevalence of severe sarcopenia among female patients with rheumatoid arthritis
Source: Sci Rep. 2021 Oct 14;11:20485. doi: 10.1038/s41598-021-99894-6 (PMC8516961; doi:10.1038/s41598-021-99894-6)
Supplement: Supplementary file 1 — Supplementary Information. [file 41598_2021_99894_MOESM1_ESM.docx]

**Serum vitamin D status inversely associates with a prevalence of severe sarcopenia among female patients with rheumatoid arthritis**

Hiroto Minamino, Masao Katsushima , Mie Torii, Wataru Yamamoto, Yoshihito Fujita, Kaori Ikeda, Emi Okamura, Kosaku Murakami, Ryu Watanabe, Koichi Murata, Hiromu Ito, Masao Tanaka, Hidenori Arai, Shuichi Matsuda, Akio Morinobu, Nobuya Inagaki and Motomu Hashimoto

**Supplementary information:**

**Supplementary Table S1. Multivariate logistic analyses with serum 25(OH)D as a continuous variable**

|  | **Multivariate** | | | | | |
| --- | --- | --- | --- | --- | --- | --- |
|  | **model 1** | | **model 2** | | **model 3** | |
| **variables** | **OR (95% CI)** | ***P* value** | **OR (95% CI)** | ***P* value** | **OR (95% CI)** | ***P* value** |
| Age (1 year) | 1.17 (1.09 - 1.25) | < 0.0001 | 1.20 (1.10 - 1.32) | < 0.0001 | 1.20 (1.10 - 1.32) | < 0.0001 |
| Body mass index (1 kg/m^2^) | 0.72 (0.62 - 0.84) | < 0.0001 | 0.76 (0.61 - 0.95) | 0.0075 | 0.76 (0.61 - 0.95) | 0.0083 |
| Serum 25(OH)D (1 ng/ml) | 0.91 (0.84 - 0.99) | 0.028 | 0.89 (0.81 - 0.99) | 0.023 | 0.89 (0.81 - 0.99) | 0.022 |
| DAS28-ESR |  |  | 1.08 (0.65 - 1.78) | 0.77 | 1.08 (0.65 - 1.79) | 0.76 |
| Stage (3, 4 vs. 1, 2) |  |  | 4.29 (1.30 - 14.1) | 0.012 | 4.31 (1.31 - 14.2) | 0.011 |
| HAQ |  |  |  |  |  |  |
| Methotrexate use |  |  | 1.75 (0.57 - 5.43) | 0.32 | 1.74 (0.56 - 5.41) | 0.33 |
| Prednisolone use |  |  | 2.80 (0.94 - 8.34) | 0.064 | 2.68 (0.85 - 8.46) | 0.092 |
| Biological agents use |  |  | 0.82 (0.28 - 2.42) | 0.72 | 0.83 (0.28 - 2.46) | 0.73 |
| MNA-SF |  |  | 0.94 (0.69 - 1.26) | 0.67 | 0.94 (0.69 - 1.26) | 0.66 |
| Osteoporosis medication (+) |  |  |  |  | 1.14 (0.39 - 3.32) | 0.82 |

**Supplementary Table S1**

Results of multivariate logistic analyses for independent variables associated with severe sarcopenia.

Model 1: adjusted for serum 25(OH)D concentration, age, and body mass index.

Model 2: Model 1 plus nutrition status (MNA-SF) and RA-related factors (DAS28-ESR, Stage, HAQ, and therapeutics (use of prednisolone, biologics, and methotrexate)). Model 3: Model 2 plus the prevalence of osteoporosis medication

Abbreviations: *RA* rheumatoid arthritis, *DAS28* disease activity score using 28 joints, *HAQ* health assessment questionnaire, *MNA-SF* Mini Nutritional Assessment Short-Form

**Supplementary Table S2. Multivariate logistic analysis of serum 25(OH)D with a cut-off value of 20 ng/ml**

|  | **Multivariate** | | | | | |
| --- | --- | --- | --- | --- | --- | --- |
|  | **model 1** | | **model 2** | | **model 3** | |
| **variables** | **OR (95% CI)** | ***P* value** | **OR (95% CI)** | ***P* value** | **OR (95% CI)** | ***P* value** |
| Age (1 year) | 1.17 (1.09 - 1.25) | < 0.0001 | 1.19 (1.09 - 1.30) | < 0.0001 | 1.19 (1.09 - 1.30) | < 0.0001 |
| Body mass index (1 kg/m^2^) | 0.72 (0.61 - 0.84) | < 0.0001 | 0.76 (0.61 - 0.95) | 0.014 | 0.76 (0.61 - 0.95) | 0.016 |
| Low 25(OH)D status  (< 20.0 ng/ml) | 3.87 (1.14 – 13.1) | 0.017 | 4.33 (1.02 - 18.4) | 0.047 | 4.34 (1.03 - 18.3) | 0.046 |
| DAS28-ESR |  |  | 1.12 (0.68 - 1.84) | 0.66 | 1.12 (0.68 - 1.85) | 0.66 |
| Stage (3, 4 vs. 1, 2) |  |  | 3.50 (1.10 - 11.2) | 0.035 | 3.51 (1.10 - 11.2) | 0.034 |
| Methotrexate use |  |  | 1.47 (0.48 - 4.52) | 0.50 | 1.46 (0.47 - 4.51) | 0.50 |
| Prednisolone use |  |  | 2.92 (0.99 - 8.60) | 0.051 | 2.79 (0.89 - 8.75) | 0.051 |
| Biological agents use |  |  | 0.81 (0.27 - 2.40) | 0.70 | 0.82 (0.28 - 2.43) | 0.70 |
| MNA-SF |  |  | 0.92 (0.68 - 1.24) | 0.59 | 0.92 (0.68 - 1.24) | 0.59 |
| Osteoporosis medication (+) |  |  |  |  | 1.14 (0.39 - 3.39) | 0.82 |

**Supplementary Table S2**

Results of multivariate logistic analyses for independent variables associated with severe sarcopenia.

Model 1: adjusted for Low 25(OH)D status (< 20.0 ng/ml), age, and body mass index.

Model 2: Model 1 plus nutrition status (MNA-SF) and RA-related factors (DAS28-ESR, Stage, HAQ, and therapeutics (use of prednisolone, biologics, and methotrexate)). Model 3: Model 2 plus the prevalence of osteoporosis medication

Abbreviations: *RA* rheumatoid arthritis, *DAS28* disease activity score using 28 joints, *HAQ* health assessment questionnaire, *MNA-SF* Mini Nutritional Assessment Short-Form

**Supplementary Table S3. The results of other covariates regarding multivariate regression analysis for RA patients with components of severe sarcopenia**

|  | **Low**  **physical performance** | | **Low**  **muscle strength** | | **Low**  **skeletal muscle index** | |
| --- | --- | --- | --- | --- | --- | --- |
| **Model/Variables** | **OR (95% CI)** | ***P* value** | **OR (95% CI)** | ***P* value** | **OR (95% CI)** | ***P* value** |
| **Model 1** |  |  |  |  |  |  |
| Age (1 year) | 1.10 (1.04 - 1.16) | **0.0004** | 1.03 (0.98 - 1.09) | 0.27 | 1.14 (1.06 - 1.22) | **< 0.0001** |
| Body mass index (1 kg/m^2^) | 1.03 (0.94 - 1.13) | 0.56 | 0.89 (0.80 - 0.98) | 0.019 | 0.59 (0.49 - 0.71) | **< 0.0001** |
| **Model 2** |  |  |  |  |  |  |
| Age (1 year) | 1.09 (1.02 - 1.16) | **0.0051** | 1.06 (0.98 - 1.14) | 0.16 | 1.18 (1.08 - 1.29) | **< 0.0001** |
| Body mass index (1 kg/m^2^) | 1.13 (0.99 - 1.28) | 0.075 | 0.95 (0.82 - 1.11) | 0.53 | 0.62 (0.49 - 0.79) | **< 0.0001** |
| DAS28-ESR | 1.35 (0.90 - 2.05) | 0.16 | 1.38 (0.84 - 2.28) | 0.20 | 0.83 (0.49 - 1.39) | 0.47 |
| Stage (3, 4 vs. 1, 2) | 1.27 (0.54 - 2.98) | 0.59 | 11.3 (4.36 - 29.5) | **< 0.0001** | 3.78 (1.33 - 10.8) | **0.010** |
| Methotrexate use | 1.20 (0.51 - 2.80) | 0.67 | 1.15 (0.42 - 3.15) | 0.78 | 1.74 (0.58 - 5.23) | 0.32 |
| Prednisolone use | 2.43 (1.05 – 5.63) | **0.036** | 0.72 (0.26 - 1.99) | 0.53 | 0.89 (0.30 - 2.65) | 0.84 |
| Biological agents use | 0.81 (0.37 - 1.78) | 0.61 | 1.09 (0.43 - 2.72) | 0.86 | 0.61 (0.23 - 1.63) | 0.32 |
| MNA-SF | 0.81 (0.63 - 1.03) | 0.079 | 0.76 (0.57 - 1.02) | 0.058 | 0.62 (0.49 - 1.21) | 0.41 |
| **Model 3** |  |  |  |  |  |  |
| Age (1 year) | 1.09 (1.02 - 1.16) | **0.0051** | 1.06 (0.98 - 1.14) | 0.13 | 1.18 (1.08 - 1.29) | **< 0.0001** |
| Body mass index (1 kg/m^2^) | 1.13 (0.99 - 1.29) | 0.060 | 0.95 (0.81 - 1.10) | 0.50 | 0.62 (0.49 - 0.79) | **< 0.0001** |
| DAS28-ESR | 1.38 (0.91 - 2.11) | 0.13 | 1.35 (0.80 - 2.26) | 0.26 | 0.84 (0.50 - 1.40) | 0.50 |
| Stage (3, 4 vs. 1, 2) | 1.25 (0.53 - 2.97) | 0.62 | 12.2 (4.56 - 32.9) | **< 0.0001** | 3.87 (1.36 - 11.0) | **0.0089** |
| Methotrexate use | 1.15 (0.49 - 2.72) | 0.74 | 1.29 (0.46 - 3.63) | 0.63 | 1.68 (0.56 - 5.06) | 0.35 |
| Prednisolone use | 2.02 (0.82 - 4.94) | 0.12 | 1.04 (0.33 - 3.26) | 0.94 | 0.76 (0.23 - 2.46) | 0.64 |
| Biological agents use | 0.84 (0.38 - 1.85) | 0.66 | 1.03 (0.40 - 2.61) | 0.96 | 0.63 (0.23 - 1.69) | 0.36 |
| MNA-SF | 0.89 (0.74 - 1.07) | 0.087 | 0.76 (0.56 - 1.02) | 0.056 | 0.62 (0.49 - 1.20) | 0.38 |
| Osteoporosis medication (+) | 1.09 (0.57 - 2.11) | 0.25 | 0.41 (0.15 - 1.16) | 0.091 | 1.52 (0.49 - 4.70) | 0.47 |

**Supplementary Table S3**Results of multivariate logistic analyses for independent variables associated with severe sarcopenia.

Model 1: adjusted for serum 25(OH)D concentration, age, and body mass index.

Model 2: Model 1 plus nutrition status (MNA-SF), and RA-related factors (DAS28-ESR, Stage, HAQ, and therapeutics (use of prednisolone, biologics, and methotrexate)). Model 3: Model 2 plus the prevalence of osteoporosis medication

Abbreviations: *RA* rheumatoid arthritis, *DAS28* disease activity score using 28 joints, *HAQ* health assessment questionnaire, *MNA-SF* Mini Nutritional Assessment Short-Form
